# Consolidated Evidence and New Frontiers of Liquid Biopsy in Lung Cancer: A Narrative Review

**DOI:** 10.3390/cells15181641

**Published:** 2026-09-10

**Authors:** Ornella Cantale, Alessandro Nepote, Elisa Lombardi, Federica Benso, Tommaso Trisciuoglio, Angela Listì, Francesca Bersani, Riccardo Taulli, Luisella Righi, Francesco Passiglia

**Affiliations:** 1 Ospedale San Luigi Gonzaga, 10043 Orbassano, Italy; ornella.cantale@unito.it (O.C.); alessandro.nepote@unito.it (A.N.); elisa.lombardi@unito.it (E.L.); 2 Ente Ospedaliero Cantonale, 6500 Bellinzona, Switzerland; 3 Translational Oncology—Paola Gilardi Lab, Department of Oncology, University of Turin, 10124 Turin, Italy; federica.benso@unito.it (F.B.); tommaso.trisciuoglio@unito.it (T.T.); angela.listi@unito.it (A.L.); francesca.bersani@unito.it (F.B.); riccardo.taulli@unito.it (R.T.); 4 Pathology Unit, Department of Oncology, University of Turin, 10124 Turin, Italy; luisella.righi@unito.it; 5 Department of Oncology, University of Turin, Ospedale San Luigi Gonzaga, 10043 Orbassano, Italy

**Keywords:** liquid biopsy, lung cancer, miRNA, ctDNA, multiomics, AI-based

## Abstract

Emerging liquid biopsy platforms are expanding frontiers in lung cancer care, facilitating real-time molecular insights and addressing some of the limitations of tissue biopsy. While standardization and sensitivity improvements are undergoing continuous refinement in those liquid biopsy techniques that are now established, several advancements are expanding the panorama of its potential future applications. This review offers an overview of the emerging uses of liquid biopsy in the field of lung cancer, with a specific focus on emerging technologies. We comprehensively reviewed the literature through PubMed and Embase, combining keywords and Medical Subject Headings (MeSH) related to small-cell (SCLC) and non-small-cell lung cancer (NSCLC) and liquid biopsy using Boolean operators. The search was limited to publications from 2017 up to 2026. Titles and abstracts underwent manual scrutiny for relevance. We critically analyzed a body of evidence assessing the potential role of liquid biopsy in common and uncommon applications, such as its combination with machine learning and AI and its integration in multiomics approaches or its usage in next-generation technologies—including label-free CTC isolation, DNA fragmentomics and end-motif profiling, four-dimensional proteomics, and unconventional biospecimens—that remain largely unaddressed in prior narrative reviews on this topic.

## 1. Introduction

Lung cancer represents the leading cause of cancer-related mortality worldwide. It is commonly classified into two main categories: non-small-cell lung cancer (NSCLC), comprising 85–90% of all lung cancer diagnoses, and small-cell lung cancer (SCLC), representing the remaining 10–15% [1,2]. Over the last 20 years, significant progress in understanding the development of NSCLC has enabled the introduction of new therapeutic strategies as immunotherapy and targeted therapies, making advanced stages curable [3]. Immunotherapy has been introduced in SCLC too, although with more modest benefits in prognosis, which currently remains poor [4]. In NSCLC, the assessment of molecular testing to identify therapeutic targets requires good quantity and quality of tissue. Tissue biopsy remains the gold standard for diagnosis and management, despite the risk of complications due to the invasiveness of the procedure. As a risk-free, repeatable procedure, liquid biopsy (LB) has been progressively integrated into clinical practice to help address these issues [5]. Indeed, LB is a minimally invasive diagnostic technique that analyses tumor-derived materials present in body fluids such as blood, saliva, urine, cerebrospinal fluid and pleural effusion. It is mostly performed in plasma, which is rich in cell-free DNA (cfDNA) and circulating tumor DNA (ctDNA), shed from tumor cells through active or passive mechanisms such as apoptosis or necrosis, respectively [6]. It was first conceptualized in 1977 and its correlation with treatment outcomes emerged early on, alongside its prognostic value [7]. The first Food and Drug Administration (FDA) approval of an LB ctDNA-based diagnostic tool occurred in 2016 for the detection of EGFR mutations after the results of the ENSURE study, which demonstrated a concordance of 76.7% between plasma and tissue-based tests in EGFR-positive patients and of 98.2% in negative tissue [8]. In advanced-stage NSCLC, where tumor burden is conspicuous and ctDNA levels are higher, LB has demonstrated strong utility on disease monitoring and identification of acquired resistance; this has contributed to the increased reliability of LB and the integration of this tool into clinical practice in this setting. Conversely, its role in the early stages is still evolving, facing the constraints of sensitivity imposed by the currently limited diagnostic tools [6]. Furthermore, LB in SCLC has been hypothesized for multiple clinical applications, including early detection, molecular subtyping, prognostic assessment, and the guidance of treatment decisions. However, current evidence is modest due to small cohorts and a lack of dedicated trials [9,10]. In addition to its established applications, a growing body of evidence supports the potential role of LB in cancer patients. These include the detection of circulating tumor cells (CTCs), extracellular vesicles (EVs), microRNAs (miRNAs), and DNA methylation, as well as combination with artificial intelligence (AI) and integration into multiomics approaches for diagnosis and monitoring purposes [11,12]. We conducted a literature review to provide a comprehensive overview of the emerging applications of LB and their significance and role in the clinical management of lung cancer, with a specifical focus on rapidly emerging technologies that are reshaping the analytical frontier of liquid biopsy. We included next-generation approaches alongside more established approaches. We tried to report the current translational maturity of each technique and clinical relevance in everyday practice.

## 2. Methods

We performed a comprehensive literature search using PubMed and Embase, using a combination of keywords and Medical Subject Headings (MeSH) related to LB, NSCLC, SCLC, CTCs, EVs, miRNA, multiomics, AI and methylation, using Boolean operators to refine the results. Specifically, we used the following combination of terms: “non-small-cell lung cancer” OR “NSCLC” AND “liquid biopsy” AND CTC OR EVs OR miRNA OR multiomics OR AI OR methylation; “small-cell lung cancer” OR “SCLC” AND “liquid biopsy” AND CTC OR EVs OR miRNA OR multiomics OR AI OR methylation; “lung cancer” AND “liquid biopsy” AND CTC OR EVs OR miRNA OR multiomics OR AI OR methylation. The literature search was restricted to studies published between 2017 and 2026, with exceptions made for pivotal studies. Studies were prioritized for inclusion based on the strength of evidence, sample size and clinical relevance. Titles and abstracts were first screened for relevance and underwent full text evaluation thereafter. Additional pertinent studies were identified through manual screening of the reference list of selected publications. The exclusion criteria included articles that did not report original data such as editorials, conference abstracts and publications not available in full text or in English.

## 3. Results

### 3.1. Circulating Tumor Cells (CTCs)

CTCs represent a pivotal frontier in the context of lung cancer [13]; a comprehensive overview of the main studies reviewed on CTCs is available in Table 1. CTCs are defined as malignant cells that shed from both the primary tumor and metastatic sites, intravasate into the bloodstream and circulate; these can be tracked as markers for non-invasive monitoring of tumor dynamics through liquid biopsy [13]. In lung cancer, CTCs embody the metastatic cascade, where epithelial–mesenchymal transition (EMT) facilitates their dissemination, allowing the evasion of *anoikis*—a form of programmed cell death triggered by detachment from the extracellular matrix—and survival in the circulatory environment [13]. These cells, often numbering fewer than one per milliliter of blood, exhibit heterogeneous phenotypes, including epithelial (EpCAM+), mesenchymal (vimentin+, N-cadherin+), or hybrid states, reflecting adaptive plasticity that correlates with aggressive disease progression, chemoresistance, and poor prognosis [14].

Therefore, CTCs in lung cancer have prognostic and predictive utility: their elevated counts precede radiological detection of metastases and stratify patients into risk categories [15]. A meta-analysis conducted in 2017 comprising 2060 patients with lung cancer showed that baseline circulating CTCs levels greater than five cells per 7.5 mL of blood were significantly correlated with OS (HR = 2.63, 95% CI [2.04, 3.39]) and PFS (HR = 3.74, 95% CI [2.49, 5.61]) [15]. The pooled OR values showed that CTCs were associated with NSCLC stage (OR = 2.11, 95% CI [1.42, 3.14]), SCLC stage (OR = 10.91, 95% CI [4.10, 29.06]), distant metastasis (OR = 7.06, 95% CI [2.82, 17.66]), lymph node metastasis (OR = 2.31, 95% CI [1.19, 4.46]), and PS (OR = 0.42, 95% CI [0.22, 0.78]) [15]. CTCs in SCLC easily exceed the 10–100 per mL due its early dissemination tendency, correlating with extensive-stage disease and therapeutic response [9,13]. Mishra A. et al. [18] proved, in a prospective cohort of 20 patients, that pretreatment DLL3 expression on SCLC CTCs predicts tarlatamab clinical benefit with 85% sensitivity and 100% specificity. Beyond mere counting, CTC clusters amplify metastatic potential up to 100-fold through enhanced stemness and immune evasion, as evidenced by murine models and clinical correlatives in advanced NSCLC cohorts [19,20,21].

Technological advancements have revolutionized CTC detection and characterization, overcoming prior limitations imposed by their rarity and phenotypic heterogeneity [22]. Immunomagnetic enrichment targeting EpCAM (e.g., CellSearch) remains the gold standard for epithelial CTCs but underestimates mesenchymal subtypes prevalent in lung cancer post-EMT [17,23]. Microfluidic devices, such as the CTC-iChip or Parsortix, leverage size-based filtration, deformability, or dielectrophoresis to achieve label-free isolation, yielding viable cells for downstream molecular profiling [22]. As an example, the TellDx CTC platform (TellBio, Inc., Archimedic, Beverly, Massachusetts) exploits the CTC-iChip technology, which combines the negative depletion of leukocytes with inertial focusing [19]. Beyond these platforms, acoustophoresis has recently gained particular traction: two-step platforms combining size-sensitive acoustic deflection with acoustic-impedance-based focusing have improved the enrichment of viable CTCs suitable for downstream culture and drug sensitivity testing, extending beyond the enumeration-only readout of CellSearch-based platforms already discussed in Table 1 [24,25].

Another proposed strategy relies on DNA aptamers, which have high affinity, stability, and the ability to recognize diverse cancer-specific biomarkers, representing an alternative to EpCAM-dependent detection. In a recent study, the use of aptamer LC 17 and LC 18 allowed heterogeneous CTC populations in lung cancer to be captured independently from EpCAM expression. In this study, elevated CTC counts were positively correlated with higher T and N stage and shorter overall survival [23].

A conceptually distinct frontier is in vivo CTC isolation, exemplified by the GILUPI CellCollector—a CE-marked, anti-EpCAM-functionalized structured medical wire inserted into a peripheral vein for direct blood-contact capture over a substantially larger sampled blood volume than standard draws. In both NSCLC and SCLC patients, this approach achieved higher CTC-positivity rates than CellSearch (58% vs. 27% in one series of 50 patients) and enabled downstream molecular characterization (EGFR, KRAS, ALK, PD-L1) directly from in vivo-captured cells [23].

High-definition imaging and RNA sequencing of single CTCs unveil actionable genomic alterations—like EGFR mutations, ALK rearrangements, or KRAS variants—mirroring primary tumor heterogeneity and enabling precision medicine [20,21]. In lung cancer trials, CTC-derived PD-L1 expression predicts immunotherapy response more dynamically than tissue biopsies, while liquid biopsy panels integrating CTCs with ctDNA enhance sensitivity for MRD detection post-resection or therapy [20,23].

### 3.2. MicroRNA (miRNAs)

MicroRNAs (miRNAs), together with long noncoding RNAs (lncRNAs), belong to the family of noncoding RNAs, which play a key role in multiple biological processes, including carcinogenesis [26,27]. A comprehensive overview of the main studies reviewed on miRNA is available in Table 2.

miRNAs are small (18–25 nucleotides), single-stranded, highly conserved RNA molecules that negatively regulate gene expression post-transcriptionally [27,30,31].

In lung cancer, miRNAs have multiple roles. They have been associated with cancer risk, particularly in Asian populations, as in the case of miR-146a rs2910164 G>C [28]. Moreover, they may serve as tools to distinguish between malignant and benign conditions [25].

Regarding their application as non-invasive biomarkers detectable through LB, miRNAs are notably actively released by cells into the extracellular environment and exhibit high stability in a wide range of biofluids—including blood, saliva, and urine—thereby enabling a reliable and quantitative assessment of their expression levels [32,33].

A recent meta-analysis showed that miRNAs have adequate accuracy for early lung cancer diagnosis [29], with a sensitivity of 0.78 (0.70–0.84) and a specificity of 0.81 (0.74–0.81), although the included studies investigated different miRNA molecules and panels. The same concept has been reinforced in the bioMILD study, where a plasma 24-microRNA signature classifier (MSC) combined with low-dose computed tomography (LDCT) was shown to increase the accuracy of lung cancer risk and mortality prediction and supported its clinical utility in the management of LDCT findings of uncertain malignancy [34,35]. A lung cancer RR increase in MSC+ compared to MSC- participants was observed in both the CTind (RR: 2.5; 95% CI: 1.4–4.32) and CT+ (RR: 2.6; 95% CI: 1.81–3.74) groups and was maintained when considering stage I or resectable tumors only. A 98% negative predictive value in CTind/MSC− and a 30% positive predictive value in CT+/MSC+ lesions were recorded. At seven years’ follow-up, MSC+ participants had a cumulative HR of 4.4 (95% CI: 3.0–6.4) for lung cancer incidence and of 8.1 (95% CI: 2.7–24.5) for lung cancer mortality [36]. Similarly, another meta-analysis reported a sensitivity of 0.76, a specificity of 0.79, and a diagnostic odds ratio (dOR) of 13.93 [26].

Moreover, a recent study investigated the role of miR-155 and miR-3196 in patients with NSCLC compared to healthy donors. Overall, the results showed that miR-155-3p was upregulated in NSCLC, whereas miR-3196 was downregulated compared to controls; the AUC values were 0.881 and 0.784, respectively. Normalization of miR-155-3p levels during cancer therapy was associated with improved OS, supporting its prognostic value and utility for treatment monitoring [27].

As extensively reported by Iqbal et al., numerous miRNA signatures have been explored to assess different aspects of lung cancer, including the distinction between adenocarcinoma and squamous cell carcinoma. Furthermore, their dysregulation has been associated with specific molecular alterations, such as KRAS or EGFR mutations [32].

### 3.3. Extracellular Vesicles (EVs)

EVs are nanosized extracellular vesicles typically ranging from 30 to 100 nm in diameter, characterized by a disc-like structure enclosed by a lipid bilayer membrane encapsulating proteins, lipids, DNAs and RNAs [37,38]. In 1987, Johnston et al. first reported that reticulocytes release small vesicles into extracellular space [35]. EVs, as non-invasive biomarkers through liquid biopsy, are one of the most vastly explored applications in lung cancer, also dependent on the predictive components embodied—miRNA, proteins, RNA and DNA [38,39]. A comprehensive overview of the main studies reviewed on EVs is available in Table 3.

Currently, two possible pathways have been theorized for the biogenesis of EVs. The endosomal process involves the inward budding of the plasma membrane to form early endosomes, which subsequently mature into multivesicular bodies. These then fuse with the plasma membrane and are released extracellularly as EVs [34]. The alternative is known as the plasma membrane pathway and hypothesizes that EVs originate through the direct outward budding of the plasma membrane [34].

Novel EV isolation strategies are increasingly moving beyond ultracentrifugation and precipitation toward label-free, high-purity, and often automated platforms. Acoustic and oscillatory nanofiltration systems, exemplified by the EXODUS (Exosome Detection via the Ultrafast-isolation System) platform, use pressure fluctuations and dual-frequency vibrations to achieve the rapid, size-based purification of small EVs from plasma with higher purity and recovery than conventional ultracentrifugation, while preserving vesicle integrity and enabling downstream molecular analyses [43]. Dielectrophoresis (DEP)-based microfluidic biosensors exploit differences in EV size, surface charge, and dielectric properties within non-uniform electric fields to selectively trap or deflect EVs without labels, offering fast processing, integration into compact chips, and compatibility with functional readouts such as impedance or optical detection [44,45]. Asymmetric flow field-flow fractionation (AF4, also termed A4F) separates EVs in a channel under a perpendicular cross-flow, resolving subpopulations by hydrodynamic size and enabling high-purity fractions suitable for proteomic, RNA, and functional studies, albeit with lower throughput and greater methodological complexity than bulk methods [46].

Tumor-derived EVs (TEX) play a multifaceted role in cancer, modulating the immune system, promoting tumor cell proliferation and facilitating metastasis [47]. EVs contribute to immunosuppression by inducing a reduction in antigen-presenting cells (APCs) and by impairing T cell and natural killer (NK) cell activity; this enables immune evasion and supports tumor growth and dissemination [47]. EVs in the tumor microenvironment (TME) carry bioactive molecules that enhance oncogenic signaling. For instance, platelet-derived EVs can induce cyclin D2 expression and activate MAPK signaling in lung cancer cells; moreover, several exosomal miRNAs, including miR-660-5p, miR-96, miR-29a, and miR-21, promote proliferation through regulatory targets and the interaction with Toll-like receptors within the TME [37]. EVs also play a pivotal role in the metastatic cascade. They promote epithelial–mesenchymal transition (EMT) through miRNAs activating STAT3 signaling or by the regulation of the E-cadherin via TGF-β pathways [44]. In addition, EVs enhance angiogenesis by transferring pro-angiogenic miRNAs that activate pathways such as JAK2/STAT3 and upregulate factors including VEGF, MMP9, and FGF2. Finally, exosomal RNA contributes to premetastatic niche formation by activating TLR3 signaling in alveolar epithelial cells, leading to chemokine production and neutrophil recruitment via NF-κB and MAPK pathways [38]. Overall, if EVs represent horizontal communicators between cells, their content could mirror the one from the cell of origin, giving rise to a new and accessible source for tumor profiling analysis [48].

EV-derived miRNAs have been detected in both systemic samples such as plasma and serum and site-specific sources, including saliva, urine, sputum, bronchoalveolar lavage fluid (BALF), and pleural effusions [45]. Several studies have identified significantly dysregulated panels of miRNAs in lung cancer patients in comparison to control samples: hsa-miR17-3p/-21/-106a/-146/-155/-191/-192/-203/-205/-210/-212/-214 [49], as well as miR-320d, -320c [40], and tumor-derived EVs miRNAs (AD-specific miR-181-5p/miR-30a-3p/miR30e-3p/miR-361-5p, and SCC-specific miR-10b-5p/miR-15b-5p/miR-320b) and serum EV miRNAs (miR-146a-5p/miR-486-5p), which can adequately work as biomarkers [39]. Overall, these miRNA-based approaches demonstrate moderate-to-good diagnostic performance in NSCLC, with reported sensitivity of approximately 67–73% and specificity of 66–80%, supporting their clinical utility for early detection and disease stratification [50]. Recent studies have also explored the role of EVs for the genetic testing of EGFR mutations in advanced lung adenocarcinoma, with high pooled sensitivity (0.82, 95% CI: 0.50–0.95) and specificity (0.95, 95% CI: 0.24–1.00) [51]. In addition, specific exosomal miRNA signatures have shown potential in predicting response to immunotherapy in advanced NSCLC. Exosomal PD-L1 (exo-PD-L1) represents a key mechanism underlying the immune checkpoint resistance in the context of immune checkpoint inhibitor (ICI) therapy [42]. It can function as a decoy by competitively binding anti-PD-L1 antibodies, thereby limiting their availability to target membrane-bound PD-L1 on tumor cells. This mechanism may contribute to the reduced therapeutic efficacy of ICIs [52,53]. The role of EVs has been investigated in SCLC, too. A recent study analyzed CTCs and EVs in 100 treatment-naïve SCLC patients, demonstrating that the overexpression of transcription factor JunB (JUNB) and C-X-C chemokine receptor type 4 (CXCR4) in EVs can distinguish patients from normal donors and that their presence in CTCs correlates with significantly poorer overall survival [41]. These results pave the way for the development of prognostic and potentially diagnostic tools in SCLC.

### 3.4. Methylation

Aberrant DNA methylation is an early and common process in carcinogenesis across multiple cancer types, including NSCLC and SCLC [54,55]. A comprehensive overview of the main studies reviewed on methylation is available in Table 4. DNA methylation consists of the covalent addition of methyl groups to the 5′ position of cytosine residues, catalyzed by DNA methyltransferases (DNMTs), and plays a pivotal role in multiple biological processes, such as chromatin architecture, X-chromosome inactivation, and genomic imprinting [56].

In biological fluids, DNA methylation may be detected through DNA fragment analysis from blood samples, but also from urine, sputum, or bronchoalveolar lavage fluid (BALF), with different sensitivity depending on the type of primary malignancy considered and the assay technique adopted [60,61]. For example, in prostate or renal cancer, DNA methylation detected through urine analysis is more specific and informative compared to results obtained from plasma analysis [62]. Similarly, in SCLC and NSCLC, DNA methylation in pleural effusion, sputum, and BALF has emerged as a possible alternative to plasma samples to distinguish malignant from benign pleural effusion when cytology is uncertain [63], to reveal early lung cancer before spreading [60], or to discriminate lung cancer from other benign diseases such as pneumonia, asthma, or sarcoidosis [61].

While its role as a potential biomarker for early carcinogenesis has been widely explored [60], DNA methylation and, more generally, epigenetic changes, including, for example, dynamic histone plasticity [63], might represent a relevant explorable tool across the natural history of cancer [64,65].

In a recent prospective translational study [55], the combined use of multiomics profiling of cfDNA methylation with extracellular vesicle (EV)-miRNAs was able to predict and identify different survival outcomes across biologically distinct NSCLC patients, maintaining temporal relevance and consistency in external cohorts.

In SCLC, a recent study adopted and analyzed genome-wide methylation through liquid biopsy to identify different subtypes of SCLC and monitor disease progression and treatment response [58].

Among the multiple genes that have been explored, CDO1 and HOXA9 have been extensively investigated in several studies [59,63,66] and have shown moderate sensitivity for early-stage cancer detection and an association with unfavorable outcomes. A recent systematic review and meta-analysis emphasized the role of CDO1 promoter methylation as a robust diagnostic tool both in blood and in alternative liquid biopsies, with a pooled diagnostic odds ratio (DOR) of 21.00 (95% CI: 14.19–31.08) and 14.52 (95% CI: 7.41–28.49), respectively [54]. The test for overall effect was highly significant in both analyses, with no statistically significant variation across sample types, confirming consistency among different biological fluids [54].

A recent methylome profiling in a longitudinal series of 22 p.G12C KRAS-mutated NSCLC patients receiving sotorasib demonstrated that methylation signature may be combined with genomic analysis to personalize therapeutic strategies for this population [67]. Indeed, NGS KRAS p.G12C VAF, alongside methylation index (MI) score, was measured across longitudinal plasma samples of such cohort. Of note, exon 2 p.G12C KRAS mutation and MI score highlighted a trend simultaneously moving forward at the first longitudinal timepoint (r = 0.68, *p* = 0.06) and at progressive disease (r = 0.87, *p* = 0.000103), indicating that the integration of genomic profiling in the longitudinal monitoring of tumor evolution can be crucial in routine practice [67].

Abnormal DNA methylation also represents a promising therapeutic target, with multiple pharmacological strategies (DNMTi, HDACi, EZH2i, PRMT5i) due to their ability to reprogram chromatin, shift cancer cell states, and indirectly restore or enhance immune responses against cancer cells [68].

Beyond conventional bisulfite- or enzymatic-based methylation assays, which were majorly adopted by the studies cited above, several next-generation approaches are emerging. Three-dimensional DNA walker nanodevices—DNA nanomachines that generate amplified electrochemical or fluorescent signals through enzyme-driven, stepwise movement along a nanostructured substrate—enable the ultrasensitive detection of methylated DNA without requiring PCR amplification, and have recently been coupled with metal–organic framework electrocatalysts to achieve rapid, low-input methylation quantification [69].

Complementary to methylation-centric approaches, cell-free DNA (cfDNA) fragmentomics has emerged as a powerful, mutation-agnostic liquid biopsy strategy. Because nuclease-mediated cleavage patterns differ systematically between malignant and non-malignant cell populations, the analysis of fragment size distribution and 4-mer end-motif composition at cfDNA fragment termini can generate diagnostic signatures even in samples with low tumor fraction. In NSCLC specifically, deep-learning classifiers integrating fragment end-motif-by-size (FEMS) features with genomic coverage have achieved AUCs up to 0.937 for lung cancer detection, with generalizability demonstrated across Korean and Caucasian validation cohorts [66]. Similar fragmentomic strategies combining methylation-based fragment size ratio (m-FSR) and end-motif distribution (m-FDEM) with multiomic integration have been proposed specifically for early-stage lung cancer screening [70], and the integration of end-motif and fragment length data with cfDNA-derived chromatin immunoprecipitation profiling (cfChIP-seq) has enabled inference of active gene expression programs directly from plasma in stage IV NSCLC patients [71].

### 3.5. AI and Multiomics

**Figure 1:**
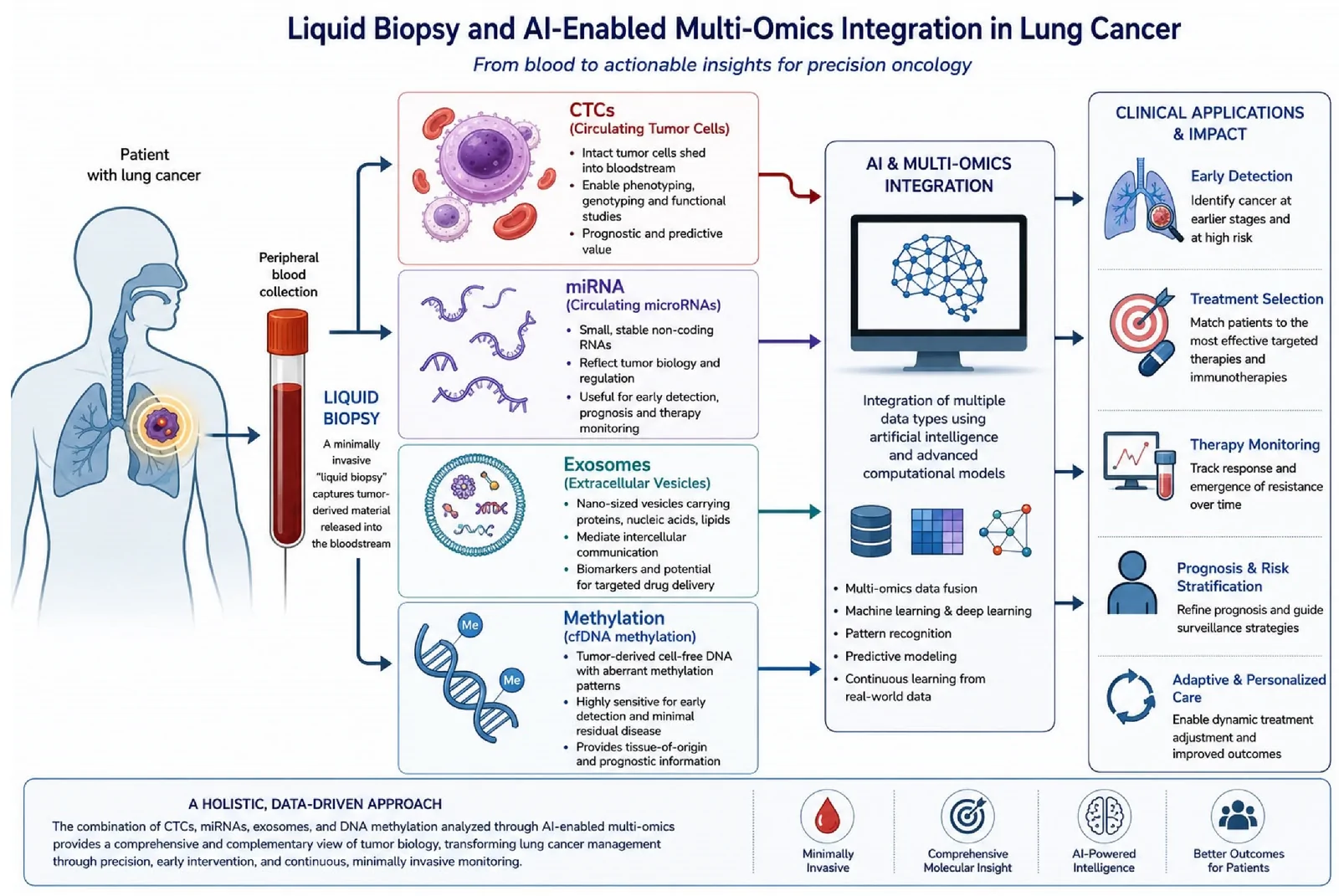
Liquid biopsy and AI-enabled multiomics integration in lung cancer.

Recent advancements in multiomics technologies and AI have dramatically reshaped the landscape of cancer research, diagnosis, and treatment, particularly in NSCLC [72]. Multiomics approaches combine complementary data layers to provide a systems-level understanding of tumor biology, while modern AI algorithms enable the analysis of these high-dimensional datasets [72], as summarized in Figure 1. Trials in this field are still lacking, although a dedicated body of literature is rapidly evolving.

Of note is the work of Wang et al. [73], who developed and validated an interpretable artificial intelligence-assisted model called PRIME (Progression Risk prediction by Interpretable Machine learning on ctDNA-MRD, Mutations, and clinical therapeutic features) onto a global dataset of 781 blood samples from stage I to III NSCLC patients. This working team found that clinical stage, pretreatment ctDNA, post-treatment MRD, blood-based Kelch-like ECH-associated protein 1 (KEAP1), serine/threonine kinase 11 (STK11), cyclin-dependent kinase inhibitor 2A (CDKN2A) mutations, and treatment modality were significantly associated with the risk of disease progression and were thereby included in the model training. The neural network (NN) model exhibited optimal prediction of treatment failure risk in the training (AUC = 0.85, 95% CI 0.81–0.89) and validation sets (AUC = 0.82, 95% CI 0.74–0.89), outperforming single liquid biopsy biomarkers and clinical therapeutic signature.

As for early detection, AI-driven multiomics integration has optimized the identification of molecular signatures associated with pre- and early malignant lesions. Multiomics biomarkers derived from genomic, epigenomic, and transcriptomic data are proving highly effective for differentiating malignant from benign nodules in high-risk populations. As reported by Ling et al. (2026) [74], combining AI with multi-layer omics has led to improvements in sensitivity and specificity for early-stage lung cancer detection, particularly when integrated with low-dose CT (LDCT) screening. Such AI-enabled frameworks efficiently extract latent biological patterns from multiomics data, supporting molecular risk stratification, histologic subtyping, and patient-specific pathway modeling to enhance early diagnosis and reduce false-positive rates in clinical screening programs [75,76,77].

In translational oncology, proteogenomic and radiogenomic models are making considerable leaps in predicting tumor aggressiveness, therapeutic resistance, and recurrence risk, being more reliable than conventional markers such as PD-L1 alone [78]. For example, Wilkins et al. (2025) emphasize that AI models integrating radiomics, pathomics, metabolomics, and immunogenomics data improve the prediction of immunotherapy response and toxicity [79]. These predictive frameworks identify key immune signatures and neoantigenic patterns that distinguish responders to PD-1/PD-L1 checkpoint inhibitors, allowing cost-effective patient selection for immunotherapy in lung cancer too [75]. In this regard, the hybrid model involving convolutional neural networks (CNNs) and recurrent neural networks (RNNs) achieves an accuracy of 97.3%, and deep neural networks (DNNs) also demonstrate efficacy in identifying subtle lung cancer patterns and outperforming existing approaches in early detection and classification [80,81].

Interpretability and transparency—crucial for clinical adoption—have been supported by Explainable AI (XAI) methods such as SHapley Additive exPlanations (SHAP) and Gradient-weighted Class Activation Mapping (Grad-CAM); these approaches assess how genomic or imaging features contribute to diagnostic or prognostic predictions, thereby improving physician trust in automated systems [82,83]. Graph neural networks (GNNs) now model protein–protein interaction networks to identify druggable molecular hubs in NSCLC, while multimodal transformers enable the cross-fusion of histopathology, radiology, and omics data for subtyping and therapy selection [80]. Federated learning frameworks, as described in multi-center collaborations, promote model generalization by harmonizing diverse datasets from different institutions without centralizing sensitive health data [81].

### 3.6. Proteomics

Proteins are the functional effectors of cellular processes and more directly reflect a tumor’s activity than genomic or transcriptomic alterations alone, since DNA-level changes do not always translate into proportional protein-level changes [82]. In lung cancer, circulating and tumor-derived proteins are therefore studied as complementary biomarkers, with the aim of capturing post-translational modifications and signaling states that nucleic acid-based assays cannot directly detect [82].

Proteomic profiling relies mainly on mass spectrometry (MS) techniques, often combined with liquid chromatography (LC). In lung cancer, MS has already been successfully applied as a diagnostic tool to predict and detect the most common NSCLC mutations and their variation along the natural history of the disease, showing high sensitivity even in the presence of low-quality tissue [83].

Back in 2014, Li et al. quantified over 4000 protein groups across matched NSCLC primary tumors, normal lung, and patient-derived xenografts, identifying metabolism-associated proteome signatures with prognostic significance on fresh tissue [84].

Since then, new techniques have emerged, such as proximity extension assay (PEA) technology (Olink), which combines antibody recognition with qPCR readout for high-specificity multiplexed quantification; and SomaScan, which uses aptamer-based (SOMAmer) probes to similar effect, revealing protein signatures not always captured by traditional immunoassays [85].

In a recent study across six prospective cohorts (LC3/INTEGRAL), profiling of over 1100 circulating proteins via the Olink platform in 731 smoking-matched lung cancer cases and controls identified 36 proteins (including CEACAM5, CA-125/MUC-16, and IGFBP-1) associated with risk of imminent lung cancer diagnosis up to three years before clinical diagnosis, predominantly implicated in invasion/metastasis, proliferative signaling, tumor-promoting inflammation, and angiogenesis [86].

MS-based proteomics itself continues to evolve toward greater depth: four-dimensional (4D) proteomics, based on trapped ion mobility spectrometry (TIMS) coupled with PASEF [87], adds ion mobility as a fourth separation dimension to conventional LC-MS/MS, enabling reproducible quantification of over 6000–7000 protein groups from minimal input in short LC gradients [87].

4D-DIA profiling has already been applied to lung cancer models, revealing interesting crosstalk between lung carcinogenesis and cardiovascular disease: in murine models, the establishment of a lung cancer environment led to significant changes in both metabolic and protein profiles within heart tissue. It has also been applied in a more clinical context to BALF, where an untargeted approach identified potentially upregulated biomarkers in NSCLC [88].

### 3.7. Emerging Biospecimens for Lung Cancer Liquid Biopsy

Apart from blood, which still represents the most common analyzed fluid for LB, new emerging biospecimens have been taken into consideration to overcome blood limitation, including the withdrawing process and the time for analysis.

Sputum, BALF, pleural effusion and urine have already demonstrated utility as alternative matrices for exosomal miRNA [40] and methylation [61,64,67,68,89,90] profiling.

Exhaled breath condensate (EBC), is a fully non-invasive, lung-specific matrix containing cell-free nucleic acids, proteins, and volatile organic compounds (VOCs). EBC is obtained during tidal breathing through the cooling and condensation of exhaled aerosol and because it is acellular and organ-proximal, it may reduce background interference from non-tumor systemic DNA sources (e.g., clonal hematopoiesis) relative to plasma. Due to its feasibility, emerging portable collection devices additionally support at-home sampling [91,92].

### 3.8. High-Molecular-Weight cfDNA and Long-Read Sequencing

Emerging frontiers in liquid biopsy for lung cancer increasingly emphasize the analysis of high-molecular-weight (HMW) cell-free DNA (cfDNA) and the application of long-read sequencing (LRS) technologies to overcome the limitations of conventional short-read approaches [93,94,95,96]. HMW cfDNA fragments—often exceeding 1–10 kb and sometimes reaching >20 kb—can originate from active release mechanisms such as necrosis, extracellular vesicles, or neutrophil extracellular traps, and may carry distinct tumor-derived epigenetic and structural information not captured by the typical ~160–180 bp apoptotic fragments [97]. Long-read platforms such as Oxford Nanopore and PacBio HiFi enable direct sequencing of these longer fragments, preserving haplotype context, structural variants, fusion genes, and native methylation patterns without PCR or bisulfite conversion, thereby enhancing detection sensitivity and biological interpretability in lung cancer liquid biopsies [93,96]. Early proof-of-concept studies in NSCLC have demonstrated that the nanopore sequencing of plasma or bronchoalveolar lavage cfDNA can accurately profile copy-number alterations, fragmentomics, and methylation signatures—even when input material is limited—and that a substantial fraction of cfDNA fragments in these samples exceed 1 kb, underscoring the untapped potential of HMW cfDNA for multimodal biomarker discovery [97].

## 4. Discussion

CTCs, miRNAs, EVs, DNA methylation signatures, and AI-based multiomics integration each offer complementary opportunities to shift lung cancer care from tissue-centric diagnostics to dynamic, blood-based precision medicine.

### 4.1. Circulating Tumor Cells (CTCs)

CTCs illustrate the translational potential of LB by providing live cellular material for phenotypic and functional analyses. Persistence or phenotypic shifts in CTCs during therapy often precede acquired resistance—for example, mesenchymal-like CTCs that upregulate AXL or MET pathways in EGFR TKI failure. The advantage of serial CTCs profiling is that it can therefore reveal clonal evolution in near real time and inform adaptive management. Preclinical interventions that target CTC-specific vulnerabilities—antibody–drug conjugates against EpCAM, nanoparticle-mediated reversal of epithelial–mesenchymal transition (EMT), bispecific T-cell engagers, and anti-cluster prophylactic approaches—demonstrate proof-of-concept for eradicating micrometastatic seeds before macroscopic relapse. Early clinical trials that use changes in liquid biopsy status (CTCs, ctDNA) to tailor dosing (for example, adaptive alectinib schedules informed by serial monitoring of ctDNA in the BFAST trial [91]) suggest improvements in PFS in selected cohorts. Nevertheless, translation to routine practice remains constrained by heterogeneous detection platforms, the absence of consensus definitions for clinically relevant CTC subsets, and limited prospective validation in early-stage disease where CTC frequency is low. The integration of single-cell multiomics and machine learning holds promise for refining predictive models and identifying actionable CTC phenotypes, but harmonized protocols and prospective clinical evaluation are still awaited.

### 4.2. MicroRNA (miRNA)

Circulating miRNAs offer technical and functional advantages as blood-based biomarkers. They are intrinsically stable in plasma, amenable to robust isolation, and retain detectability after long-term frozen storage, supporting retrospective biomarker discovery and multi-center validation. A singular contribution comes from Balzano et al. [92], who analyzed eight miRNA species extracted from plasma at different timepoints: fresh, 6-month, and 12-month stored at −80 °C, demonstrating high stability and a long-frozen half-life. Functionally, circulating miRNA signatures reflect tumor-derived regulatory programs related to proliferation, immune modulation, and treatment resistance; panels of miRNAs have shown potential for early detection, prognostication, and prediction of therapy response. Nevertheless, some key limitations must be addressed, including platform-dependent variability in quantification, the need for large, well-phenotyped cohorts to establish clinically meaningful thresholds, and the challenge of discriminating tumor-derived miRNAs from those originating in non-malignant tissues or systemic responses. Addressing these issues via standardized assays and prospective validation will be crucial for clinical translation.

### 4.3. Extracellular Vesicles (EVs)

As for EVs, clinical implementation is hindered by several limitations. Among these are the lack of standardized isolation methods, the biological heterogeneity that complicates the discrimination of tumor-derived vesicles, and the absence of clinically validated biomarkers. Furthermore, the pre-analytical variability and the limited availability of large prospective clinical studies restrict their current applicability in routine practice [93].

Future research will investigate the potential therapeutic role of EVs as anticancer drug delivery vehicles, especially for agents with low solubility and limited off-target delivery. EVs can be loaded with therapeutics directly (e.g., electroporation, extrusion, sonication) or indirectly (e.g., donor cell engineering) [94]. New strategies include ligand/antibody conjugation, modulation of glycan interactions, and engineering of integrins to redirect tissue tropism. Advances in microfluidics and nanotechnology further enhance EV stability, functionalization, and tracking. Beyond drug delivery, EVs show promise in immuno-oncology: dendritic cell-derived EVs (DEX) and TEX can present tumor-associated antigens and can modulate immune responses [95]. Therefore, engineered EVs are expected to emerge as a versatile platform for targeted drug delivery and cancer immunotherapy, potentially inducing durable immune responses and improving therapeutic outcomes [50].

### 4.4. Methylation

Epigenetic profiling by ctDNA methylation analysis represents another robust avenue for early detection and tumor characterization. Methylation-based assays can detect cancer-specific patterns that are present even when mutation-based signals are sparse, potentially enabling screening across tumor types and sensitive monitoring of minimal residual disease. Yet, DNA methylation tests face important specificity challenges: non-malignant processes such as chronic inflammation, infections, comorbid conditions, and physiological states (for example, pregnancy) can partially mimic the same methylation status, increasing the false positive rate [96].

New perspectives and opportunities are related to the analysis of the whole methylome rather than the analysis of multiple single-gene promoters for a specific disease, enabling a more holistic view of disease etiology and allowing more comprehensive population screening [98].

To summarize, the analysis of DNA methylation through LB could play a key role in multiple cancer detection, including SCLC and NSCLC, in monitoring disease progression, and in identifying resistant cancer cell populations. It may also evolve into a theranostic tool by enabling the prediction of response to epigenetic-targeted therapies. The emergence of new strategies to overcome the technical limitations of traditional assays is enabling a shift from single-locus methylation biomarkers toward genome-wide, structurally and fragmentomically informed liquid biopsy signatures, and warrant dedicated evaluation in future NSCLC/SCLC biomarker studies.

### 4.5. AI and Multiomics

Across all modalities, the integration of AI and multiomics is the logical next step to convert complex biomarker data into clinically actionable intelligence. Multi-modal datasets can capture the multifaceted biology of lung cancer and enable personalized risk stratification, treatment selection, and adaptive monitoring. However, technical hurdles (data harmonization, batch effect correction, modality-specific normalization), regulatory and ethical considerations, costs, and the current lack of extensive prospective validation limit the current clinical adoption. To address these limitations, collaborative initiatives advocate for standardized data pipelines, the integration of real-world evidence, and the implementation of digital clinical decision support systems (CDSSs) capable of continuous learning from new data streams [99].

Looking forward, the convergence of AI, single-cell technologies, and multiomics profiling heralds a paradigm shift toward precision medicine in lung cancer. As integrative computational frameworks mature, they promise to transform research findings into clinically actionable strategies, enabling early and minimally invasive detection, rational treatment selection, and adaptive monitoring of therapeutic response. Ultimately, the synergy between AI and multiomics sciences is moving lung cancer care from population-based paradigms toward personalized, data-driven models that capture the molecular complexity of each patient’s disease and continuously refine care through iterative learning [75,84,100].

Despite a significant body of evidence on liquid biopsy in lung cancer, we are still far from translating it into clinical practice. Across different techniques and modalities, translational maturity varies substantially [15,18,23,24,25,28,29,35,36,56,79,88,101,102,103,104,105,106,107,108,109].

The bioMILD 24-miRNA/LDCT signature has reached prospective screening evaluation with population-level mortality data (NCI Phase 4); CDO1/HOXA9 methylation panels and the SCLC-DMC classifier sit at Phase 3 (retrospective longitudinal validation); while the emerging technologies discussed in this review—3D DNA walkers, cfDNA fragmentomics/end-motif profiling, 4D-proteomics, label-free CTC platforms, and novel EV isolation methods—remain predominantly at the preclinical/early clinical assay stage (Phase 1–2).

None of the new approaches discussed here has yet reached Phase 5 (demonstrated population-level impact on cancer-specific mortality). More prospective trials, specifically designed with endpoints tailored to liquid biopsy, are needed for the field to fully enter this new era. Current evidence differentially supports liquid biopsy across distinct clinical applications: prognostic stratification (CTC counts, miRNA signatures), monitoring of acquired resistance and treatment response (serial ctDNA/CTC dynamics, exo-PD-L1), and—more preliminarily—early detection/screening (bioMILD) and minimal residual disease surveillance. Screening and diagnostic use-cases in early-stage disease remain the most evidence-limited, consistent with the sensitivity constraints discussed earlier in this review [6].

Practical considerations beyond analytical performance are critical determinants of clinical implementation. Turnaround time (TAT) varies substantially by assay design, laboratory logistics, and reporting workflow: proof-of-concept Oxford Nanopore long-read cfDNA workflows have generated interpretable copy-number and fragmentomic results from plasma or urine in less than 24 h [110,111], whereas clinical plasma NGS testing in NSCLC has historically required a median of approximately 7–13 days from blood draw or laboratory receipt to final reporting [112,113]. In a routine clinical laboratory, established assays may be easier to standardize and deploy at scale than emerging long-read, fragmentomic or EV-based approaches; for example, the FDA-cleared CellSearch system provides CTC-enumeration results within approximately two days after sample receipt, although its cleared indications are metastatic breast, prostate and colorectal cancers rather than lung cancer. Regulatory maturity also differs markedly across liquid biopsy modalities [111]. The cobas^®^ EGFR Mutation Test v2 is FDA-approved as a plasma-based companion diagnostic for selected EGFR alterations in metastatic NSCLC, including acquired EGFR T790M for the identification of patients eligible for osimertinib after progression on EGFR-TKI therapy, whereas most EV, cfDNA-fragmentomic, methylation and long-read sequencing applications remain investigational and require prospective analytical and clinical validation before broad routine adoption [8].

We attempted to assign evidence level to some of the techniques discussed to identify their level of maturity, as shown in Table 5. To systematically classify them, we adopted the National Cancer Institute’s five-phase framework for biomarkers, which has been designed to validate biomarkers from early preclinical management to population-level cancer control. We did not report any technique/assay in phase 5, since the only acceptable application in routine clinical practice is the usage of ctDNA for detecting actionable mutations at diagnosis in the absence of tissue and resistant mutations during treatment.

This review has several limitations. As a narrative review, it is subject to potential selection bias in the literature discussed, despite the structured search strategy described in the Section 2. The studies cited are highly heterogeneous in design and sample size, limiting direct cross-study comparison. The fast-evolving nature of the field makes it difficult to immediately capture ongoing innovations, and even more difficult to predict the translational status of the techniques and modalities discussed, making it hard to determine in advance which of them will be introduced into clinical practice.

## 5. Conclusions

In sum, each liquid biopsy component offers distinctive strengths—CTCs for live cellular phenotyping and therapeutic targeting, miRNAs for stable circulating indicators, EVs for integrated molecular cargo and delivery potential, and methylation patterns for sensitive detection—while AI-enabled multiomics integration provides the computational framework to synthesize these signals. Realizing this potential will depend on methodological standardization, rigorous prospective validation, and close collaboration between translational researchers, clinicians, regulators, and industry. If these challenges are addressed, liquid biopsy paradigms could markedly improve early detection, personalize treatment selection, and enable adaptive, minimally invasive surveillance—thereby reducing the morbidity and mortality associated with metastatic lung cancer.

## Figures and Tables

**Table 1:** Emerging applications of CTCs in lung cancer.

Author, Years	Study Design	Patients (n)	Diagnosis Stage	Source	Technique	Main Objective	Key Findings	Limitations
Zhu et al., 2024 [14]	Retrospective, Single center	8000	NSCLC, SCLC All stages	Blood	CellSearch, dPCR	Correlations between clinical/pathological features and CTCs	NSCLC (1) adenocarcinoma: CD68 and P40 expression were independent factors associated with CTCs. (2) squamous: tumor size, pleural indentation, and air bronchogram were independent factors affecting CTCs. SCLC no correlations between clinical features and CTCs levels	Study design, Asian population only, single method of CTC isolation
Xu et al., 2017 [15]	Meta analysis	2060	NSCLC SCLC All stages	Blood	CellSearch, RT-PCR, RTQ-PCR, LT-PCR, IF, ICC, ISET	Prognostic value of CTCs in all settings	CTCs are associated with poor OS (HR = 2.63, 95% CI [2.04, 3.39]) and PFS (HR = 3.74, 95% CI [2.49, 5.61]) CTCs were associated with NSCLC and SCLC stage (OR = 2.11, 95% CI [1.42, 3.14]) (OR = 10.91, 95% CI [4.10, 29.06]), distant mets (OR = 7.06, 95% CI [2.82, 17.66]), lymph node mets (OR = 2.31, 95% CI [1.19, 4.46]), and performance status (OR = 0.42, 95% CI [0.22, 0.78])	Univariable data included heterogeneity among studies, methods, cut-off values, study design and selection bias
Li et al., 2021 [16]	Retrospective, single center	345	NSCLC stage I–IIIA	Blood	BEADS, CytoploRare Kit qPCR/RT-PCR	Prognostic value of pre-operative CTCs	Preoperative CTCs concentration is an independent prognostic factor for NSCLC patients (HR 5.489; [95% CI: 2.660–11.326, *p* < 0.001])	Study design, Asiatic population only, single method of CTC isolation and no value for postoperative CTCs
Ntzifa et al., 2024 [17]	Prospective, single center	30	NSCLC EGFR+ stage IV	Blood	ISET, Parsortix™ (ANGLE plc, Guildford, UK) TRIZOL	Monitoring evolution of the disease	Variance in concordance among CTCs and ctDNA Detection of mechanisms of resistance	Sample size, limited external application, no validation cohort
Mishra et al., 2026 [18]	Prospective, single center	20	ES-SCLC	Blood	CTC-iChip, Chromium GEM-X Single Cell 3′ Kit v4, P2 XLEAP-SBS	Predictive value of DLL3 on CTCs in ES-SCLC treated with tarlatamab	(1) In DLL3-positive >25% CTCs, 11 of 11 (100%) DLL3^Pos^ patients had clinical benefit (6 PR and 5 SD) from tarlatamab (2) CTFs accumulation may predict severe CRS and the need for inpatient hospitalization	Sample size; no brain mets pts

Abbreviations: CTCs: circulating tumor cells; CTF: circulating tumor fragment; ES-SCLC: extended-stage small-cell lung cancer; ICC: immunocytochemistry; IF: immunofluorescence; NSCLC: non-small-cell lung cancer; mets: metastases; PCR: polymerase chain reaction; PFS: progression-free survival; OS: overall survival; PR: partial response; pts: patients; RT/RTQ/LT-PCR: reverse transcription/real time quantitative/long-target PCR; SCLC: small-cell lung cancer.

**Table 2:** Emerging applications of microRNAs in lung cancer.

Author, Years	Study Design	Patients (n)	Diagnosis Stage	Source	Technique	Main Objective	Key Findings	Limitations
Wang et al., 2020 [28]	Meta analysis	6506 cases 6576 controls	NSCLC and SCLC	Blood	PCR-RFLP, TaqMan, MALDI-TOF-MS, PCR-HRMA PCR	Predictive role of miR-146a polymorphism and lung cancer susceptibility	miR-146a rs2910164 was significantly associated with increased lung cancer susceptibility across several genetic models	Selection bias, selected populations, high heterogeneity (I^2^)
Fehlmann et al., 2020 [25]	Retrospective Multi-center	3046	NSCLC and SCLC and controls	Blood	PAXgene blood miRNA Kit miRNA Complete Labelling and Hyb Kit (Agilent Technologies)	Predictive role of miRNAs for detecting lung cancer in an extended cohort of pts vs. controls (nontumor lung diseases, diseases not affecting the lung, unaffected control participants).	A 15-miRNA signature identified lung cancer with 91.4% accuracy, 82.8% sensitivity and 93.5% specificity. A 14-miRNA signature identified patients with lung cancer from patients with nontumor lung diseases with 92.5% accuracy, 96.4%, 88.6% specificity. A 14-miRNA signature identified patients with early-stage lung cancer from all individuals without lung cancer with 95.9% accuracy, 76.3% sensitivity and 97.5% specificity	Study design, methodological limitations in measuring microRNAs; not directly applicable for clinical use
Shen et al., 2022 [29]	Meta analysis	1039 (7 studies for microRNAs)	Early-stage NSCLC	Blood	PCR	Early diagnostic tool of microRNAs in early-stage lung cancer	miRNAs have adequate accuracy for early lung cancer diagnosis, with a sensitivity of 0.78 (0.70–0.84) and a specificity of 0.81 (0.74–0.81)	Heterogeneous assay; information bias majority of studies retrospective.
Mlika et al., 2024 [26]	Meta analysis	22,224	Lung cancer, All stages	Blood, pleural effusion, sputum, bronchoalveolar lavage	QT-PCR, RT-PCR, dPCR	Diagnostic performance of miRNA in lung cancer	Pooled sensitivity 0.76, specificity 0.79 and AUC 0.859, suggesting good diagnostic accuracy	Significant heterogeneity; mean quality score of studies included was very low
Alexandre et al., 2025 [27]	Observational	136 pts and 64 healthy donors	NSCLC, including SCC and LUAD All stages	Blood	RT-qPCR	microRNA as a diagnostic, prognostic and monitoring biomarkers in NSCLC	miR-155-3p was upregulated and miR-3196 downregulated in NSCLC; high miR-155-3p was associated with shorter OS. During systemic cancer therapy circulating levels of both miRNAs tended to normalize. Normalization of miR-155-3p levels was associated with improved OS.	Small sample size, low sensitivity and/or specificity for each marker, lack of a standardized measurement

Abbreviations: AUC: area under the curve; LUAD: lung adenocarcinoma; NSCLC: non-small-cell lung cancer; PCR: polymerase chain reaction; pts: patients; OS: overall survival; RFLP: restriction fragment length polymorphism; SCC: squamous cell carcinoma; SCLC: small-cell lung cancer.

**Table 3:** Emerging EV applications in lung cancer.

Author, Years	Study Design	Patients (n)	Diagnosis Stage	Source	Technique	Main Objective	Key Findings	Limitations
Qi et al., 2019 [37]	Translational study	40 pts/40 healthy control	NSCLC	Plasma (blood)	EV isolation, qRT-PCR, colony formation, Transwell assays, luciferase assay, Western blot, xenograft model	To determine the role of exosomal miR-660-5p in NSCLC progression and identify its target gene	miR-660-5p is elevated in NSCLC plasma/EVs, promotes proliferation, migration, invasion and tumor growth	Small cohort, no stage information, no survival analysis, limited mechanistic exploration
Zhang et al., 2019 [38]	Observational, Biomarker study	72 pts/ 30 healthy control	NSCLC Liver cancer Pancreatic cancer	Bone marrow and blood	EV isolation (ultracentrifugation), TEM, NTA, miRNA microarray, RNA sequencing, qRT-PCR, Western blot	To elucidate the intercellular communication between hypoxic BMSC-derived EVs and cancer cells and their role in promoting metastasis and EMT	Hypoxic BMSCs release EVs enriched with miR-193a-3p, miR-210-3p, and miR-5100, which activate STAT3 signaling in cancer cells to drive EMT. A combined panel of these three miRNAs showed high diagnostic accuracy (AUC 0.8717) for detecting lung cancer mets	Complex and controversial roles of specific miRNAs (like miR-193a-3p and miR-210-3p) which can act as either oncogenes or tumor suppressors depending on the context
Peng et al., 2020 [40]	Observational retrospective exploratory biomarker study	9 pts: 5 with PR, 4 with PD, 7 healthy controls	Advanced NSCLC	Plasma (blood)	Ultracentrifugation, TEM, small-RNA next-generation sequencing (Illumina HiSeq4000)	To investigate whether plasma-derived exosomal microRNAs could serve as non-invasive biomarkers for predicting and monitoring the efficacy of ICIs in pts with advanced NSCLC	Patients with NSCLC exhibited distinct plasma exosomal microRNA signatures. Among these, hsa-miR-320d, hsa-miR-320c, and hsa-miR-320b emerged as promising biomarkers for predicting response to ICIs in advanced NSCLC. Furthermore, a reduction in the expression of the T-cell inhibitory microRNA hsa-miR-125b-5p during treatment was associated with enhanced T-cell activity and may indicate a favorable response to ICIs	Small sample size, no direct comparison with established biomarker (e.g., PD-L1 expression or TMB)
Jin et al., 2017 [39]	Case–control diagnostic study for testing validation	46 pts (26 in the testing cohort and 20 in the validation cohort)/42 healthy controls; symptomatic cohort: 60 suspected cases (47 cancers and 13 healthy)	NSCLC stage I: LUAD and SCC	Blood	Ultracentrifugation for total EVs; antiEpCAM magnetic bead-based immunoaffinity capture of tumor-derived EVs, miRNA sequencing (Illumina HiSeq), miRNA quantification (TaqMan qPCR-RT-PCR for validation)	To identify tumor-derived exosomal miRNAs able to discriminate LUAD from SCC and support non-invasive early NSCLC diagnosis	miRNA panels showed AUCs of 0.899 for NSCLC, 0.936 for LUAD, and 0.911 for SCC; miR-181b-5p, miR-30a-3p, miR-30e-3p, and miR-361-5p were LUAD-specific; miR-10b-5p, miR-15b-5p, and miR-320b were SCC-specific	Stage I only; validation in advanced stages and other biofluids is needed; weak EpCAM signal in controls was possible; non-standardized isolation methods; further refinement of biomarker combinations, accuracy, and sensitivity is required
Papakonstantinou et al., 2025 [41]	Prospective/observational study	100 chemotherapy-naïve SCLC pts for CTC analysis; 58 pts with SCLC plus 10 healthy donors for EVs analysis	SCLC, predominantly ES-SCLC (86%)	Blood and plasma	Exosomal JUNB/CXCR4/PD-L1 protein assessment by immunoblot/Western blot; small-RNA library preparation and miRNA sequencing (Ion Torrent)	To evaluate JUNB, CXCR4, and PD-L1 in CTCs and plasma EVs, and to assess the combined diagnostic/prognostic utility of these analytes in SCLC	JUNB and CXCR4 were highly prevalent in CTCs; exosomal JUNB and CXCR4 were increased in pts compared with controls and showed discriminatory potential; exosomal CXCR4 was associated with CTC presence and phenotypes; JUNB/CXCR4-positive CTCs correlated with poorer OS; this represents a first integrated CTC–EVs approach in SCLC	Low CTC recovery rate with Ficoll; unequal sample sizes for CTC and EVs analyses (100 vs. 58), affecting correlation and survival analyses; exoMIR analysis was performed in a very small subgroup; larger cohorts and validation in other tumors are required
De Miguel Perez et al., 2022 [42]	Translational study with a retrospective cohort and a prospective validation cohort	33 pts in the retrospective ICI cohort; 39 patients in the prospective PROLUNG cohort (24 Pembro + Doce, 15 Doce)	Advanced/metastatic NSCLC	Blood (timepoints: baseline and every 9 ± 1 weeks	Ultracentrifugation, NTA, TEM, Western blot	To assess whether EV PD-L1 dynamics predict durable response, PFS, OS in patients with NSCLC treated with ICIs	EV PD-L1 increased in non-responders; ΔEV PD-L1 outperformed tissue PD-L1 for durable response prediction (AUC approximately 74–75% vs. 63–64%); increased EV PD-L1 identified non-responders with 73% sensitivity and 61% specificity; reduced EV PD-L1 was associated with longer PFS and OS; tissue PD-L1 was not predictive	Tissue PD-L1 data were missing for part of cohort B; sample size was moderate; comparison with previous studies is limited by different EV isolation and measurement methods; radiomics analysis was exploratory and requires further validation

Abbreviations: anti-EpCAM: anti Epithelial-Cell Adhesion Molecule; BMSCs: bone marrow-derived mesenchymal stem cells; Doce: docetaxel; EMT: epithelial–mesenchymal transition; ICIs: immune check-point inhibitors; LUAD: lung adenocarcinoma; NSCLC: non-small-cell lung cancer; NTA: Nanoparticle Tracking Analysis; mets: metastases; PCR: polymerase chain reaction; Pembro: pembrolizumab; EV: extra-vesicles; PFS: progression-free survival; OS: overall survival; PD: progressive disease; PD-L1: programmed-death ligand 1; PR: partial response; pts: patients; SCC: squamous cell carcinoma; SCLC: small-cell lung cancer; TEM: Transmission Electron Microscopy.

**Table 4:** Emerging applications of methylation in lung cancer.

Author, Years	Study Design	Patients (n)	Diagnosis Stage	Source	Technique	Main Objective	Key Findings	Limitations
Ooki et al., 2017 [57]	Retrospective	Training cohort: 90 validation cohort: 43 serum analysis in 43 and 42 controls	NSCLC including LUAD and SCC early-stage (I-II)	Blood, pleural effusion and ascites	(Q)-MSP	To identify a methylated gene panel for early detection and prognostic stratification in NSCLC	Six-gene panel: CDO1, HOXA9, AJAP1, PTGDR, UNCX, MARCH11. In serum, sensitivity was 72.1% and specificity 71.4%; subjects with *HOXA9* methylation showed poor outcomes	Study design, selection bias for the methylations considered, underpowered considering sample size and variables included. Modest diagnostic performance
Gao et al., 2026 [54]	Meta-analysis	7 studies; 655 lung cancer pts and 402 controls	NSCLC (all histologies) SCLC	Blood, urine, sputum	Q-MSP Q-PCR	Diagnostic value of CDO1 promoter methylation in lung cancer	CDO1 promoter methylation showed a pooled sensitivity of 0.72 and specificity of 0.89 for blood-based liquid biopsies, and 0.66 and 0.87 for nonblood specimens. The overall diagnostic odds ratio was 19.13 (95% CI: 13.53–26.84), with positive and negative likelihood ratios of 7.73 (95% CI: 5.77–10.35) and 0.32 (95% CI: 0.26–0.39)	Few studies included and mostly case controls, low statistical power
Onieva et al., 2026 [55]	Prospective, longitudinal	79	NSCLC Stage IV	Blood	EM-seq	To identify molecular subtypes in NSCLC associated with immunotherapy outcome	Multiomics Factor Analysis derived clusters demonstrated consistent survival stratification in external cohorts, particularly in cluster MDC- T2	Lack of a validation cohort with paired multi omics profiling, cfMeth validation performed on tissue and not on liquid biopsy, possibly introducing biological discrepancies
Heeke et al., 2024 [58]	Retrospective	179	SCLC	Blood	PCR RRBS	DNA methylation-based classifier (SCLC-DMC) to distinguish SCLC subtypes.	Tumor and cfDNA methylation classified SCLC into biologically and clinically relevant subtypes and may allow longitudinal tracking of subtype evolution	Some key technical parameters were not assessed (RNA/DNA quality), lack of a validated assay with strict analytical criteria; sample size
Yang et al., 2018 [59]	Prospective	50	NSCLC, Stage I	Blood	QMSP	Diagnostic value of cfDNA methylation	Identification of a panel of 8 genes with 72% sensitivity and 91% specificity	Very small cohort; control group limited to inflammatory pseudotumor; low sensitivity for individual genes; needs validation in screening-like populations

Abbreviations: LUAD: lung adenocarcinoma; NSCLC: non-small-cell lung cancer; PCR: polymerase chain reaction; pts: patients; SCC: squamous cell carcinoma; SCLC: small-cell lung cancer.

**Table 5:** Overview of translational maturity of mentioned modalities/studies according to the National Cancer Institute’s 5-phase framework.

Phase	Description	Modalities/Studies Discussed in the Manuscript
Phase 1	Preclinical exploratory	3D DNA walkers; 4D-proteomics (PASEF); novel EV isolation methods (EXODUS, dielectrophoresis, AF4); CTC aptamers
Phase 2	Clinical assay and validation	End-motif profiling/fragmentomics in NSCLC (Choi et al. [114], AUC 0.937); GILUPI CellCollector; exo-PD-L1 as a predictive ICI biomarker (De Miguel Perez et al. [42])
Phase 3	Retrospective longitudinal	CDO1/HOXA9 methylation (Ooki et al. [57], Gao et al. [54]); SCLC-DMC classifier (Heeke et al. [58]); Fehlmann et al. [25] miRNA panel (91.4% accuracy)
Phase 4	Prospective screening	bioMILD study—24-miRNA panel + LDCT, with 7-year follow-up data and cancer-specific mortality HR already reported in the text [35]
Phase 5	Cancer control (population impact)	—

## Data Availability

No new data were created or analyzed in this study. Data sharing is not applicable.
